# Calcineurin *tax-6* regulates male ray development and counteracts with *kin-29* kinase in *Caenorhabditis elegans*

**DOI:** 10.1080/19768354.2019.1687584

**Published:** 2019-11-16

**Authors:** Seung Hyun Kim, Hana Jung, Joohong Ahnn, Sun-Kyung Lee

**Affiliations:** a Department of Life Sciences, Hanyang University, Seoul, Republic of Korea; b Research Institute for Natural Sciences, College of Natural Sciences, Hanyang University, Seoul, Republic of Korea

**Keywords:** *Caenorhabditis elegans*, calcineurin, salt inducible kinase (SIK), *tax-6*, *kin-29*

## Abstract

Phosphorylation is one of the critical protein modifications, which can lead to changing the activity of the proteins and regulating a variety of biological processes. Therefore, it is essential to properly maintain the phosphorylation level on proteins by balancing the activity of kinases and phosphatases. In this study, we report that calcineurin, a serine/threonine phosphatase, counteracts with a salt inducible kinase (SIK) to control male tail development in *Caenorhabditis elegans*. The counteracting regulation is cell lineage-dependent; the number of defective rays from T lineage in animals lacking calcineurin *tax-6* is decreased by knock-down of SIK *kin-29*. This result is in contrast with the knock-down of bone marrow protein (BMP) receptor kinase *sma-6*, which slightly aggravates the T lineage defect. Also, *sma-6* knock-down results in modest defect in ray 1 of V5 lineage in the absence of *tax-6* activity. Finally, knock-down of a tyrosine phosphatase *cdc-25.3* does not affect the defective ray phenotype of calcineurin *tax-6 loss-of-function(lf)* mutants. Altogether, these results suggest that balanced phosphorylation mediated by *tax-6* and *kin-29* is required for proper development of T lineage rays, and *tax-6* and *sma-6* may function in a parallel pathway in the developmental process of V5 lineage ray 1. This study emphasizes the elaborated developmental process of male ray formation, in which carefully coordinated expression of various genes is essential.

## Introduction

*Caenorhabditis elegans* are hermaphroditic nematodes and reproduce primarily by self-fertilization. However, *C. elegans* males also infrequently occur at a rate of 0.1∼0.2%, as a result of fertilization of gametes, which spontaneously lost X chromosome due to non-disjunction of X chromosome homologs during meiosis (Emmons 2005). The existence of male allow outcrossing, which significantly enhances the species genetic diversity (Barrière and Félix 2005). Male worms develop the distinctive tail structure, which is essential in male mating. The fan-like male tail has 9 pairs of rays, numbered rays 1–9 from anterior to posterior, covered within the fan cuticle, which play a pivotal role in male mating by sensing the contact of hermaphrodite mates. Although the rays share a similar structure, each has specific properties in development and function. For example, rays 1, 5, and 7 senses dorsal contact, whereas rays 2, 4, and 8 detect ventral contact (Liu and Sternberg 1995). Also, rays 7, 8, and 9 are essential for turning around hermaphrodites after contact, while rays 5, 7, and 9 are required for the timing of sharp ventral turns (Lints and Emmons 1999). Development and function of the rays are crucial in male mating behavior (Barr 2006). Therefore, what determines *C. elegans* ray structure and how it is regulated have mainly been examined to help understand genetic interactions underlying the ray developmental procedures.

The rays arise from three different lateral hypodermal blast cells of V5, V6, and T, which are differently programmed to develop. V5 gives rise to the most anterior ray 1, expressing the Hox gene *mab-5* in a repetitive on-and-off cycling mode (Salser and Kenyon 1996). *mab-5* is also activated in V6 lineages by the caudal homolog *pal-1*, activating *egl-5* and the bHLH transcription factor *lin-32*, which are necessary to establish the rays 2, 3, 4, 5 and 6 (Kenyon 1986; Zhao and Emmons 1995; Ferreira et al. 1999; Hunter et al. 1999). Cells in T lineage asymmetrically divide, and some of them finally become rays 7, 8, and 9. The process is dependent on Wnt ligand LIN-44, which plays a role in the proper orientation of cell divisions, while uncharacterized gene *mab-19* is essential in the development of T rays, but neither of V5 nor V6 (Sutherlin and Emmons 1994; Herman et al. 1995). These studies indicate that specific preprogrammed gene activation defines the identity of each ray. However, it is still mostly unknown what genes participate in the ray determining genetic program.

Calcineurin is a calcium-dependent serine/threonine phosphatase, which plays a critical role in various calcium-dependent signaling pathways (Liu et al. 2005). It consists of catalytic subunit A and a regulatory subunit B. Upon binding of calcium-bound calmodulin to subunit A, subunit B is released, and the autoinhibitory domain of subunit A frees from the active site. Subunit A becomes active (Rusnak and Mertz 2000). In *C. elegans*, *tax-6*, formerly known as *cna-1(calcineurin a-1)*, encodes calcineurin subunit A and *cnb-1(calcineurin b-1)* encodes subunit B. *tax-6 loss-of-function(lf)* mutants are small, and defective in chemotaxis and thermotaxis (Kuhara et al. 2002). The *cnb-1*null mutants have thinner cuticle surface, smaller sperm, smaller brood size, and defects in movement and egg-laying (Bandyopadhyay 2002). On the other hand, *tax-6 gain-of-function(gf)* mutants exhibit abnormal enteric muscle contraction and high sensitivity to serotonin-induced egg-laying (Lee et al. 2004; Lee et al. 2005). In males, *tax-6* is highly expressed in tail neurons and rays (Hu 2006). Both *tax-6(lf)* and *(gf)* mutants show low efficiency in male mating behaviors, including clumsy responding to hermaphrodites. Although these reports strongly suggest *tax-6* may function in structural integrity of ray, it has not been studied whether *tax-6* is required explicitly for ray development.

In this study, we investigated the ray structure in *tax-6(lf)* mutant animals. We found that the lack of *tax-6* affect the development of all rays, while the defect in T lineage rays 7, 8, and 9 is most severe. Although a serine/threonine kinase *kin-29(lf)* mutants exhibited little ray defect, RNAi treatment against *kin-29*, which is previously reported to counteract *the tax-6* activity (Singaravelu et al. 2007), suppressed the defect rate in T lineage of *tax-6(lf)* mutant. In contrast, RNAi to a protein tyrosine phosphatase *cdc25.3*, which is critical in the development of hermaphrodite germline, did not. On the other hand, feeding RNAi against BMP receptor type 1B *sma-6*, which is crucial in ray development (Krishna et al. 1999), slightly aggravated the defect in T lineage.

Interestingly, both *tax-6(lf*)and *sma-6(lf)* mutant animals were little defective in ray 1, but knock-down of *sma-6* on the lack of *tax-6* resulted in approximately 20% defect rate. These results indicate that calcineurin phosphatase *tax-6* is required for normal development of rays, differently interacting with two different types of kinases, *sma-6*, and *kin-29* in different cell lineage. This study provides insight as to how phosphorylation mediated by kinases and phosphatases regulate ray development in blast cell lineage-dependent mode.

## Materials and methods

### Strains and worm maintenance

All worms investigated in this study were obtained from CGC (Caenorhabditis Genetics Center) and maintained according to the standard protocols (Brenner 1974).

### Feeding RNAi

Bacteria containing each RNAi plasmid clone were cultured overnight). Each RNAi bacterial culture was seeded in an NGM plate containing 100 μg/ml of ampicillin and 1mM isopropyl-β-D-1-thiogalactopyranoside (IPTG). The seeded plates were incubated at room temperature overnight to induce the expression of RNAi clone. Synchronized worms were allowed to feed on RNAi bacteria, and F3 generation progenies were examined to study.

### Observation of male reproductive phenotypes

Worms used in this study were in *him-10(e1511)* background. Males are collected from the plate by washing with M9 buffer and then placed on a 3% agar pad. 2% levamisole was added on the agar pad to paralyze worms. After putting a cover glass on the agar pad, worms were observed 400× magnification using a Zeiss Axio Imager A1 microscope, and images were captured using an Axiocam camera and AxioVision software. The number of fused, lost, or malformed rays were counted as a defect, and defect rate was calculated by the number of defective rays divided by the total number of rays.

## Results and discussion

**Figure 1.**
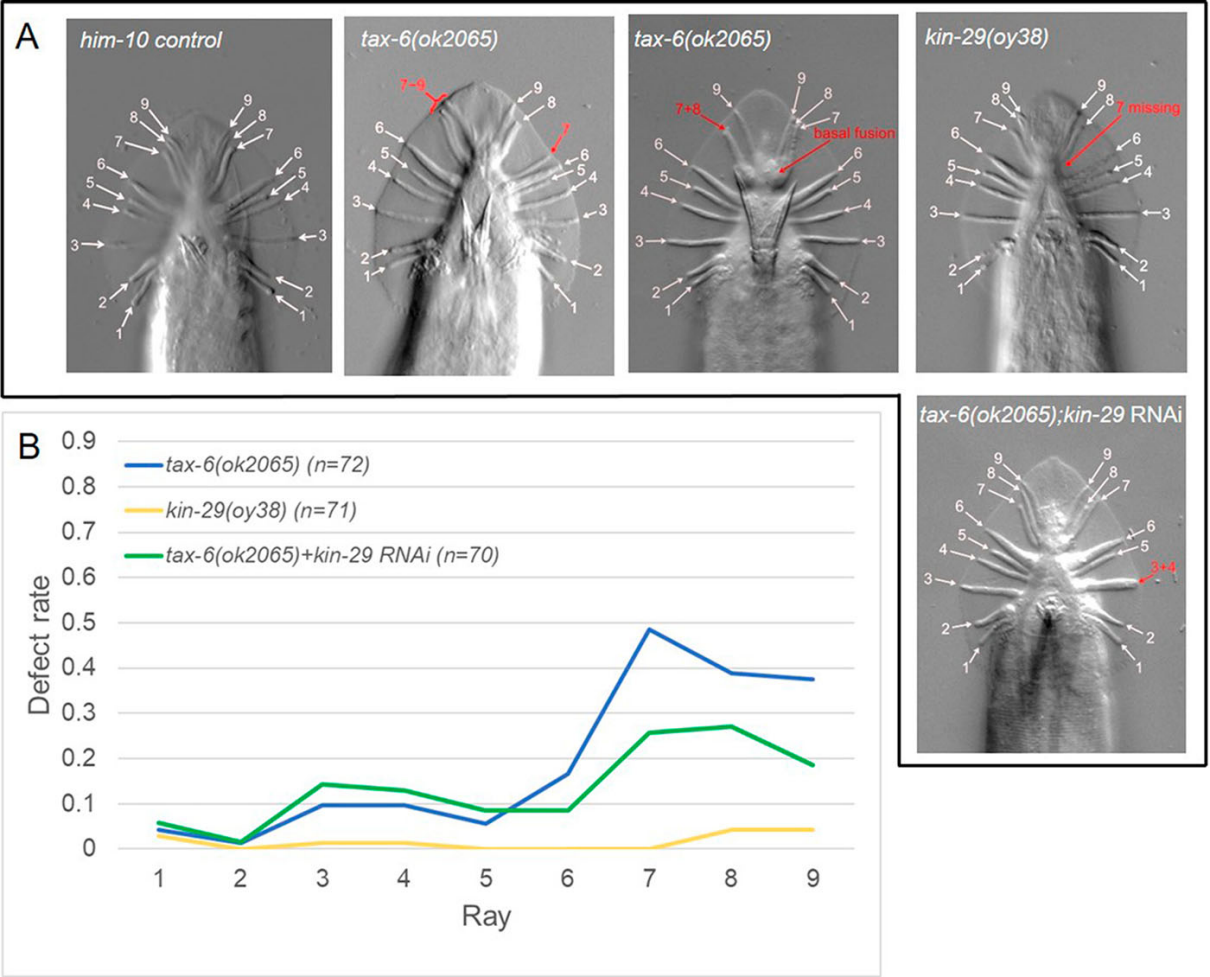
*tax-6* and *kin-29* in ray development. Representative phase-contrast images of male rays in *him-10* control, *tax-6(ok2065lf) kin-29(oy38lf)*, and *tax-6(lf);kin-29 RNAi* are shown (A). The defect rate was calculated by dividing the number of worms having fused, missing, mislocated, and/or malformed rays by the total number of observed worms, and is plotted (B). The defect in ray 6–9 in *tax-6(ok2065)* was suppressed by kin-29 RNAi, whereas that in ray 3–5 was enhanced.

Calcineurin *tax-6* is previously reported to be expressed in male rays and required for male mating behaviors in *C. elegans* (Hu 2006). However, it has been unknown how *tax-6* affect ray function. To see whether *tax-6* directly affect ray development, we set out to examine ray structure in *tax-6(lf)* mutant animals. We crossed *tax-6(ok2065)* with *him-10(e1511)*, to obtain *tax-6(lf)* in *him-10* mutant background to produce many male progenies. Approximately 40–50% of 72 observed *tax-6(lf)*worms showed defective rays at the position of 7, 8, and 9, which are T lineage (Figures 1 and 4). Also, about 15% of ray 6 was defective, although *tax-6* is reported not to be expressed in ray 6 (Hu 2006). Modest defect in ray 6 where *tax-6* expression is seemingly low in adult stage suggest that *tax-6* may be expressed and function in the early developmental stage or development of ray 6 may be influenced by neighboring rays. In contrast, less than 10% was defective in rays 1–5. Especially, rays 1 and 2 showed little defect in *tax-6(lf)*. Altogether, these data indicate that *tax-6* is necessary to develop normal rays, and its effect is predominant in T lineage rays.

Calcineurin *tax-6* is a serine/threonine protein phosphatase. Thus high phosphorylation level on its substrate proteins may be responsible for ray defect observed in *tax-6(lf)* mutant animals. Previously, SIK *kin-29* was reported to physically and genetically interact with *tax-6*, acting downstream of *tax-6* to regulate various phenotypes such as body size, egg-laying, and sensory neuronal activity (Singaravelu et al. 2007). Therefore, we asked the question of whether *kin-29* interacts with *tax-6* in male ray development. To knock-down the expression of *kin-29*, we fed *tax-6(lf)* in *him-10* mutant background worms with RNAi bacteria against *kin-29*. The RNAi treatment to *kin-29* suppressed the profound defect of T lineage rays exhibited in *tax-6(lf)*, approximately half, but slightly enhanced the defect rate in rays 3, 4, and 5 (Figures 1 and 4). To ask a question of whether *kin-29* itself functions in ray development, we examined rays in *kin-29(oy38lf)*. Interestingly, *kin-29(lf)* mutant animals showed little ray defect in overall (Figure 1B). This result is consistent with the previous report (Maduzia et al. 2005). These data suggest that *kin-29* activity would counteract with *tax-6* in ray development, possibly sharing common substrates, then downstream of *tax-6*.

**Figure 2.**
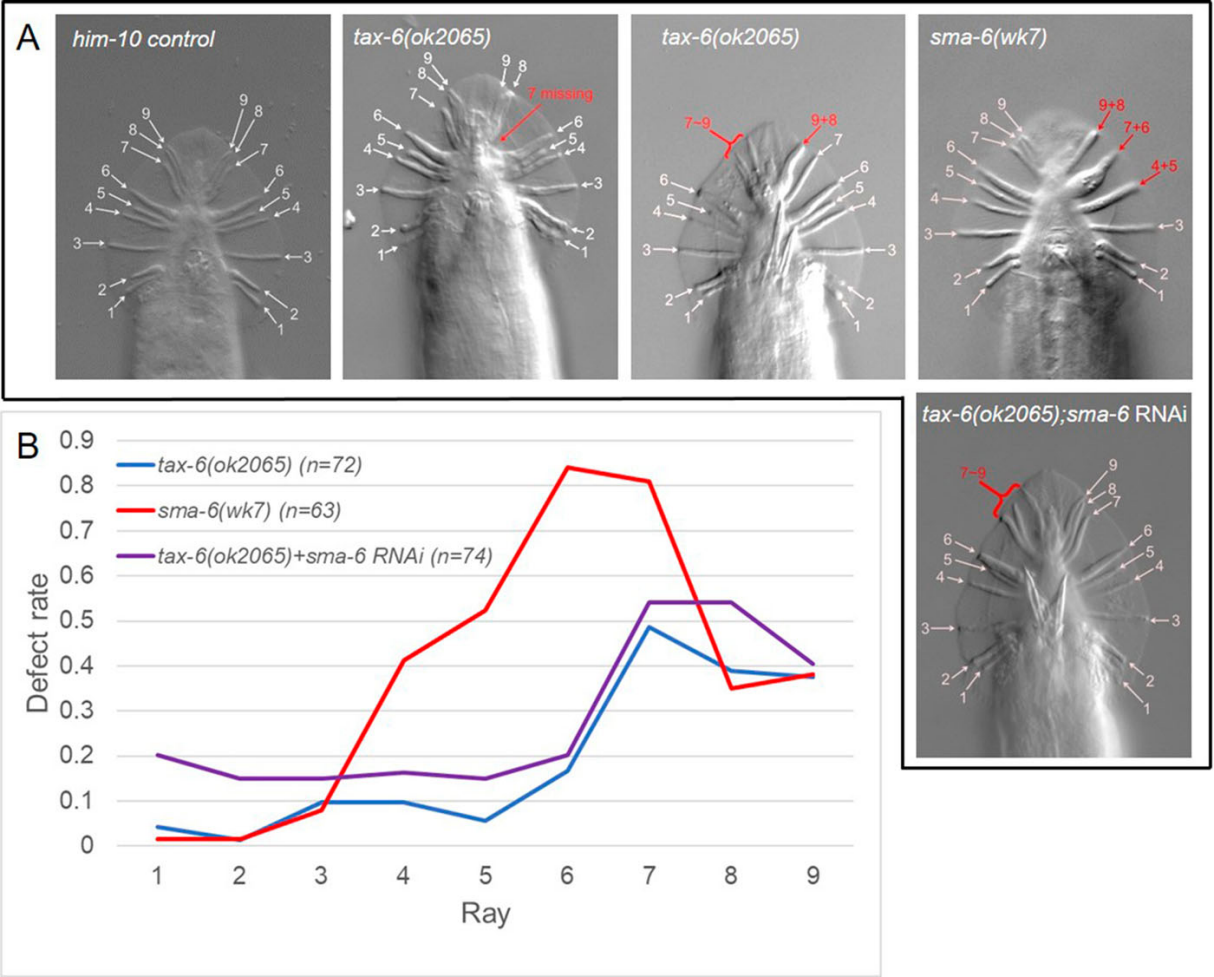
*tax-6* and *sma-6* in ray development. Representative phase-contrast images of male rays in *him-10* control, *tax-6(ok2065lf)*, *sma-6(wk7lf)*, and *tax-6(lf);sma-6 RNAi* are shown (A). The defect rate was calculated by dividing the number of worms having fused, missing, mislocated, and/or malformed rays by the total number of observed worms, and is plotted (B). The high defect rate in ray 7–9 in *tax-6(ok2065)* was not suppressed by *sma-6 RNAi*, but the defect rate in ray 1–5 in *tax-6(ok2065)* was enhanced by *sma-6 RNAi*.

BMP signaling pathway is one of the crucial genetic networks regulating male ray development in *C. elegans*. Worms lacking regular activity of BMP receptor type 1B *sma-6*, a membrane serine/threonine kinase, develop malformed male tail structure resulting in abnormal mating behavior (Krishna et al. 1999). We examined the ray structure of *sma-6(wk7lf)* mutant animals. As previously reported, many of the mutant animals showed severe malformation in their male tails. We scored individual ray defects and found that about 80% of *sma-6(lf)* worms had defects in rays 6 and 7 (Figure 2B). They also showed more than 30% defect rate in rays 4, 5, 8, and 9. These data indicate that *sma-6* functions in development mainly of V6 and T lineage rays. To investigate genetic interactions between *tax-6* and *sma-6*, we treated *tax-6(lf)* in *him-10* mutant background worms with RNAi bacteria against *sma-6.* In these worms, the overall pattern of rays defect was closer to *tax-6(lf)* than to *sma-6(lf)*, and the defect rate in rays 4–9 from V6 and T lineage was similar to that of *tax-6(lf)*, not to *sma-6(lf)*. Interestingly, in the ray 1 of V5 lineage, *tax-6(lf)* treated with *sma-6* RNAi showed approximately 20% of defect rate, in contrast to *tax-6(lf)* or sma*-6(lf)* single mutants which were almost normal in ray 1 (Figures 2 and 4). These data suggest that *tax-6* and *sma-6* may interact differently in different cell lineage rays, and *tax-6* may be downstream of *sma-6* in T and V6 lineage, but they are partially in parallel in V5 lineage. *sma-6* is reported to likely phosphorylate SMAD transcription factors, which are significant substrates for BMP receptor kinase (Wang 2005). In mammals, BMP signaling is dampened by protein phosphatase 2A(PP2A), directly dephosphorylating SMAD (Bengtsson et al. 2009). It is unknown that PP2A functions counteracting *sma-6*, as well as *tax-6*, the PP2B in *C. elegans*, which does not seem to counteract with *sma-6*.

**Figure 3.**
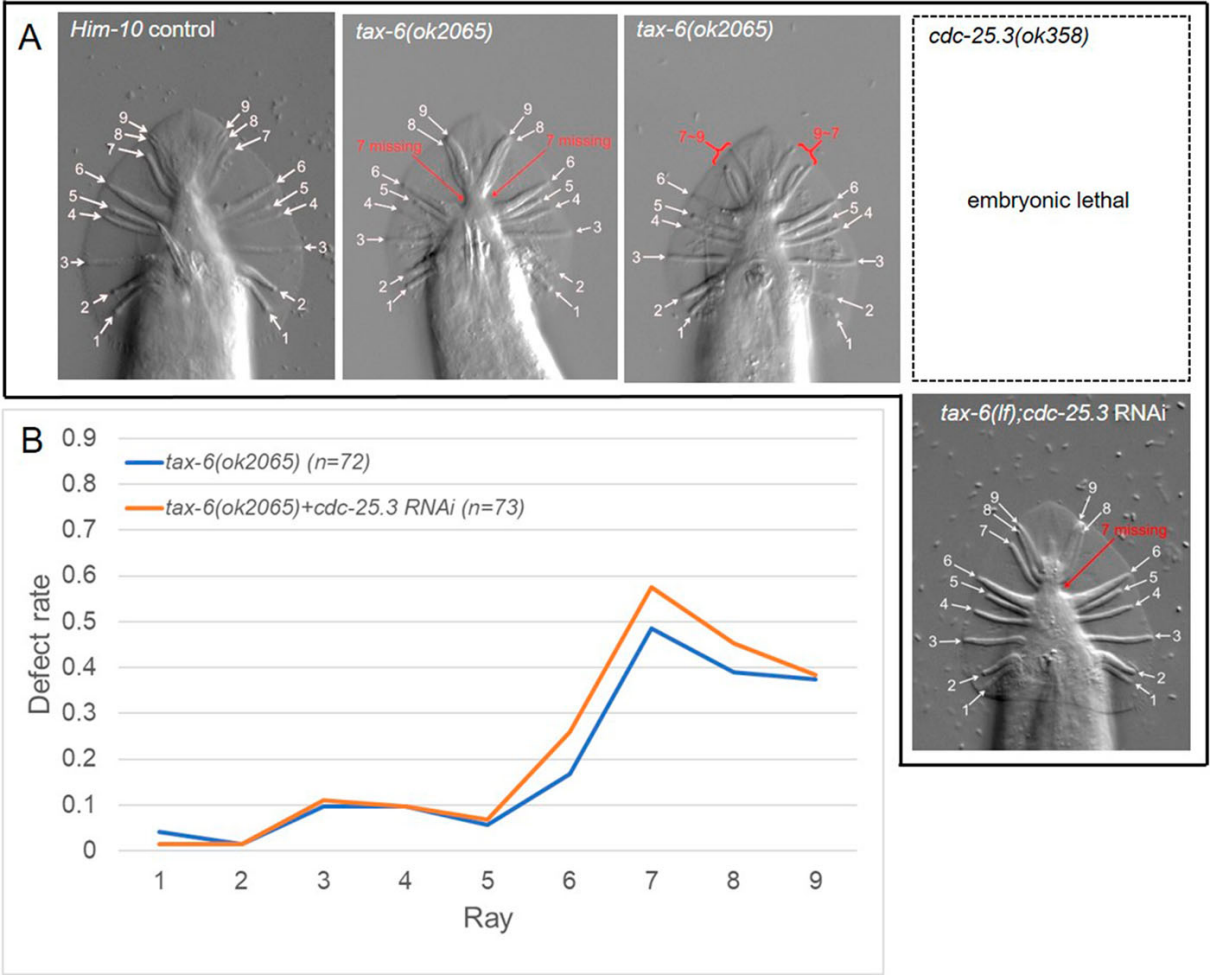
*tax-6* and *cdc-25.3* in ray development. Representative phase-contrast images of male rays in *him-10* control, *tax-6(ok2065lf)*, and *tax-6(lf);cdc-25.3 RNAi* are shown (A). Defect rate was calculated by dividing the number of worms having fused, missing, mislocated and/or malformed rays by the total number of observed worms and is plotted (B). While *cdc-25.3(ok358)* was severe embryonic lethal, there was little effect of *cdc-25.3 RNAi* on *tax-6* mutant.

**Figure 4.**
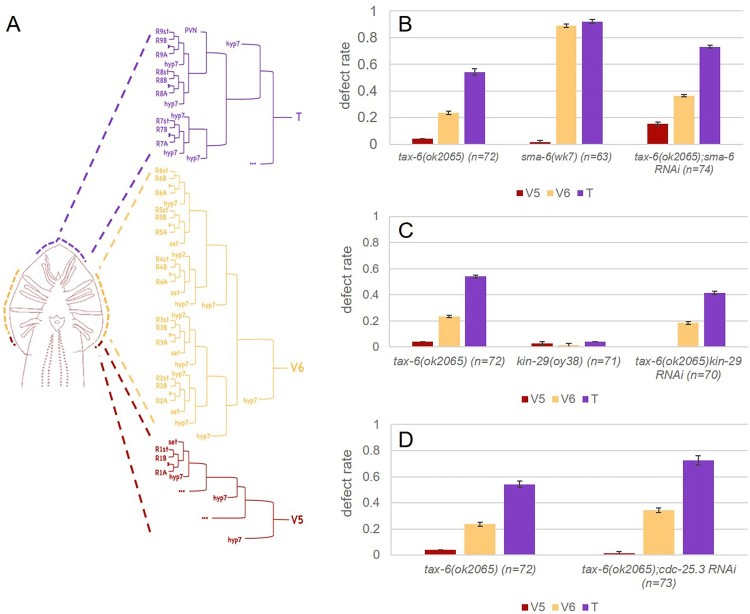
Defect rates in V5, V6, and T cell lineages of the male ray. (A) Cell lineages of male rays. Ray 1 comes from V5, and Ray 2–6 and Ray 7–9 from V6 and T, respectively. (B) The defect rate of V5, V6, and T lineages in *tax-6(ok2056)*, *sma-6(wk7),* and *tax-6(ok2065);sma-6 RNAi*. *sma-6 RNAi* enhances the ray defects of all lineages in *tax-6(ok2065)*, while *sma-6 lf* mutants almost exclusive shows ray defects in only V6 and T lineages. (C) The defect rate of V5, V6, and T lineages in *tax-6(ok2056)*, *kin-29(oy38)*, and *tax-6(ok2065);kin-29 RNAi*. *kin-29 RNAi* suppresses the ray defect of V6 and T lineages, but not V5. *kin-29 lf* mutants have normal rays. (D) The defect rate of V5, V6, and T lineages in *tax-6(ok2056)*, and *tax-6(ok2065); cdc-25.3 RNAi*. *cdc-25.3* knock-down aggravates the ray defects of V6 and T.

Because we found that a cytoplasmic SIK *kin-29* and a plasma membrane kinase *sma-6* seem to interact with calcineurin *tax-6* in slightly different modes, we decided to test a phosphatase, which is unlikely to counteract with *tax-6*. Therefore, we treated *tax-6(lf)* in *him-10* background with *cdc-25.3*, a tyrosine phosphatase, which is required for normal germline development. As expected, RNAi treatment to *cdc-25.3* did not alter much of ray defect in *tax-6(lf)*, although *cdc-25.3(ok358lf)* mutant animals showed severe embryonic lethality in hermaphrodites. However, *cdc-25.3 RNAi* slightly aggravated defects only in rays 6, 7, 8, and 9 (Figures 3 and 4). This result indicates that *cdc-25.3* might be in parallel with *tax-6* in T lineage, but is unlikely to be much effective in male tail development in overall.

*tax-6* has been reported to be expressed in neurons and supporting cells in male rays and is essential for normal male mating behavior (Hu 2006). In this study, we investigated the function of *tax-6* in ray development. We found that *tax-6* is critical for normal ray development, including ray 6, in which *tax-6* is hardly expressed (Hu 2006). Especially, rays 7, 8, and 9 in T lineage are mainly affected by the lack of *tax-6* activity. However, rays from V6 lineage seem to also need *tax-6* for normal development because of two reasons; First, all V6 rays exhibited low but similar defect rates, and second, those defect rates increased by RNAi treatment to the counteracting kinase *kin-29.* Also, there is a possibility that *tax-6* may function in Ray 1 from V5 in parallel with other genes such as *sma-6*, a membrane serine/threonine kinase with high substrate specificity (Vogt et al. 2014). In our research, *cdc-25.3* did not affect much ray defect phenotype in *tax-6(lf)* but increased the defect rate a bit in rays of T lineage. Both *cdc-25.3(lf)* and RNAi treatment to the tyrosine phosphatase result in the germline phenotype with embryonic lethality, so it was not feasible to obtain viable males to observe ray development in these worms (Figure 3).

Notably, the rays at the border between two different cell lineages tend to show defect together. For example, ray 6 is from V6 lineage, but its defect rate is relatively higher than other V6 rays despite no presumable expression of *tax-6*, in the worms in which the defect rate in T lineage is high. Also, ray 2 neighboring ray 1 from V5 tends to follow the fate of ray 1. These results indicate that the development of individual rays is mostly affected by neighboring developmental environments, as well as genetic deposition determined by cell lineages.

**Figure 5.**
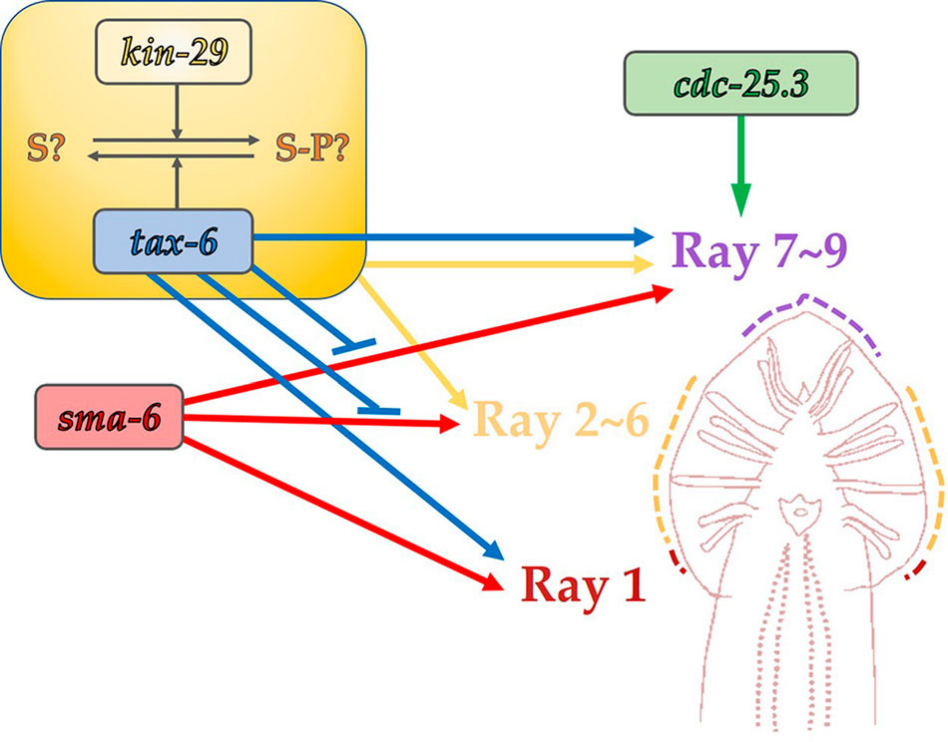
Effects of *tax-6*, *sma-6*, *kin-29*, and *cdc-25.3* on ray development. The loss of *tax-6* activity affects the development of rays from all of V5, V6 and T lineages. *sma-6* regulates V6 and T lineage rays, and *tax-6* may inhibit *sma-6* regulation on V6 and T lineage development. Counteracting with *tax-6, kin-29* regulates the development of rays 2–9, which are V6 and T lineages. *cdc-25.3* may be in parallel to *tax-6* in the development of rays 7–9 of T lineages.

**Figure 6.**
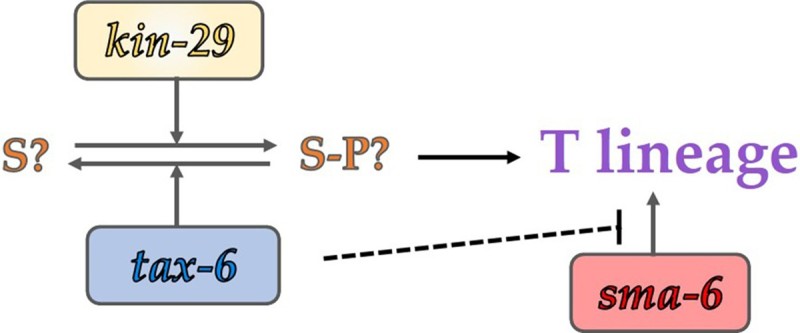
Genetic network model regulating T lineage. S is an unknown phosphorylation substrate for *tax-6* and *kin-29*. Phospho-S (S-P) positively regulates ray development in T lineage, in the pathway *tax-6* phosphatase and *kin-29* kinase balance phosphorylation level on the substrate S. *sma-6* is a direct regulator on T lineage, and *tax-6* may suppress the downstream of *sma-6*.

In this study, we report the ray development phenotype of *tax-6(lf)* mutant animals and genetic interaction between some kinase and phosphatase genes of *kin-29*, *sma-6*, and *cdc-25.*3 (Figures 5 and 6). According to our data, *tax-6* regulates ray development mainly of T lineage, also affecting V5 and V6 lineages. *kin-29*/SIK counteracts with *tax-6* activity in T lineage development, because knock-down of *kin-29* in *tax-6 lf* mutants suppresses developmental defects in T lineage, although the loss of *kin-29* shows little effect in ray development. Therefore, we propose a novel regulatory pathway of tax-6/calcineurin and kin-29/SIK, which functions in T lineage ray development. *sma-6*/BMP receptor kinase also regulates T lineage as well as V6 lineage. The loss of *tax-6* in the knock-down of *sma-6* partially restores impaired rays in T and V6 lineage, suggesting *tax-6* may suppress *the sma-6* pathway in ray development. *sma-6* also seems to be in parallel with *tax-6* in regulation of V5 lineage. *cdc-25.3*/tyrosine phosphatase regulates T lineage at least in parallel with *tax-6*, while it is unknown whether its activity is required for ray development. This study provides helpful information to understand the genetic interaction between kinases and phosphatases in tissue development at the organismal level and reinforce the intriguing features of male ray development regarding differential gene regulation of different blast cell lineages in *C. elegans*.
